# Nutrient limitation suppresses the temperature dependence of phytoplankton metabolic rates

**DOI:** 10.1038/s41396-018-0105-1

**Published:** 2018-04-25

**Authors:** Emilio Marañón, María P. Lorenzo, Pedro Cermeño, Beatriz Mouriño-Carballido

**Affiliations:** 1 Departamento de Ecología y Biología Animal, Universidade de Vigo, 36310 Vigo, Spain; 2 Instituto de Ciencias del Mar, Consejo Superior de Investigaciones Científicas, Passeig Maritim de la Barceloneta 37-49, 08003 Barcelona, Spain

**Keywords:** Microbial ecology, Climate-change ecology

## Abstract

Climate warming has the potential to alter ecosystem function through temperature-dependent changes in individual metabolic rates. The temperature sensitivity of phytoplankton metabolism is especially relevant, since these microorganisms sustain marine food webs and are major drivers of biogeochemical cycling. Phytoplankton metabolic rates increase with temperature when nutrients are abundant, but it is unknown if the same pattern applies under nutrient-limited growth conditions, which prevail over most of the ocean. Here we use continuous cultures of three cosmopolitan and biogeochemically relevant species (*Synechococcus* sp., *Skeletonema costatum* and *Emiliania huxleyi*) to determine the temperature dependence (activation energy, *E*_a_) of metabolism under different degrees of nitrogen (N) limitation. We show that both CO_2_ fixation and respiration rates increase with N supply but are largely insensitive to temperature. *E*_a_ of photosynthesis (0.11 ± 0.06 eV, mean ± SE) and respiration (0.04 ± 0.17 eV) under N-limited growth is significantly smaller than *E*_a_ of growth rate under nutrient-replete conditions (0.77 ± 0.06 eV). The reduced temperature dependence of metabolic rates under nutrient limitation can be explained in terms of enzyme kinetics, because both maximum reaction rates and half-saturation constants increase with temperature. Our results suggest that the direct, stimulating effect of rising temperatures upon phytoplankton metabolic rates will be circumscribed to ecosystems with high-nutrient availability.

## Introduction

Temperature is a master variable that controls biological activity through its effect on metabolic rates [1–3]. Within the temperature range of normal activity, metabolic rates increase with temperature according to the Boltzman–Arrhenius function:

1 $$R = ae^{ - E\mathrm{a}/kT}$$

where R is the mass-specific metabolic rate (in units of time^−1^), *k* is the Boltzmann’s constant (8.62 × 10^−5^ eV K^−1^), *T* is temperature in K, *a* is a normalization constant, and *E*_a_ is the activation energy (eV), a measure of how strongly temperature affects the metabolic rate. Increasing temperature accelerates enzymatic reactions by increasing the proportion of molecules that have sufficient kinetic energy to react [4, 5]. The fundamental nature of this thermodynamic mechanism explains that *E*_a_ of basal metabolic rate (maintenance respiration) takes relatively similar values (0.6–0.7 eV) across all organisms from microbes to plants and animals [2]. The temperature dependence of metabolic and growth rates can also be expressed by Van’t Hoff’s *Q*_10_ factor:

2 $$Q_{10} = \left( {R_2/R_1} \right)^{10/(T_2 - T_1)}$$

where *R*_2_ and *R*_1_ are the rates measured at temperatures *T*_2_ and *T*_1_, respectively. *E*_a_ values of 0.6 and 0.7 eV correspond approximately to *Q*_10_ values of 2.2 and 2.6, respectively. The temperature dependence of metabolic rates is one of the foundations of the metabolic theory of ecology (MTE), which provides a unifying framework for the prediction of ecological processes at multiple levels of organization from individuals to ecosystems [3].

Phytoplankton contribute nearly half of the annual global primary production and are major drivers of biogeochemical cycling, sustaining the food webs of most marine ecosystems and contributing to climate regulation through the uptake and sequestration of atmospheric CO_2_ [6, 7]. Mean sea surface temperature is projected to increase between 1 and 3°C by the end of this century, with the strongest warming in tropical and subtropical regions [8]. Warmer temperatures will likely cause, particularly in low-latitude, open-ocean regions, a reduction in phytoplankton productivity, as a result of enhanced thermal stratification and lower nutrient supply from sub-surface waters [9, 10]. However, this indirect effect could be counterbalanced by the direct, stimulating effect of increasing temperature upon phytoplankton growth [11–14].

Ocean ecosystem models typically use *Q*_10_ values between 1.88 and 2, based on Eppley’s data compilation [15], to parameterize the relationship between temperature and maximum phytoplankton growth rate [13, 16, 17]. Importantly, these Q_10_ values, as well as more recent estimates of the temperature dependence of phytoplankton growth [18, 19], are all based on measurements from nutrient-saturated, batch cultures, in which nutrient concentrations are typically 2–3 orders of magnitude higher than those found even in coastal, nutrient-rich waters. Yet, both experimental [20, 21] and observational [22] studies with natural communities have found reduced sensitivity of phytoplankton metabolic rates to temperature under conditions of low nutrient availability. Interpretation of these results, however, is not straightforward, due to confounding factors such as shifts in species composition across environmental gradients and also because ever-changing growth conditions during short-term, batch experiments prevent populations from attaining full physiological acclimation. Thus, the temperature dependence of phytoplankton metabolic rates under conditions of steady-state, nutrient-limited growth remains unknown. This is a major knowledge gap, because phytoplankton experience chronic nutrient limitation and sustain persistently slow growth rates in more than 80% of the global ocean [23–25]. In addition, major oligotrophic regions such as the subtropical gyres are expanding [26] and becoming more nutrient-impoverished [27] as a result of climate warming.

To determine the temperature dependence of phytoplankton metabolism under conditions of steady-state nutrient limitation, we measured *E*_a_ of photosynthesis and respiration in nitrogen-limited continuous cultures of three widely distributed and biogeochemically significant species (the diatom *Skeletonema costatum*, the coccolithophore *Emiliania huxleyi*, and the cyanobacterium *Synechococcus* sp.). Our results show that nutrient limitation suppresses the temperature dependence of metabolic rates, which means that the direct response of phytoplankton primary production to increasing ocean temperatures will differ fundamentally among ecosystems with different nutrient availability.

## Materials and methods

Chemostats and experimental setup—We maintained monospecific cultures of the diatom *Skeletonema costatum* (strain CCAP 1077/1 C), the coccolithophorid *Emiliania huxleyi* (strain CCMP 371) and the cyanobacterium *Synechococcus* sp. (strain PCC7002) under nitrogen-limited continuous growth using a Sartorius Biostat Bplus bioreactor. The bioreactor was equipped with two 2-L, double-walled borosilicate culture vessels and an integrated thermostat system with circulation pump that allowed precise (0.1 °C) control of growth temperature. Cultures were aerated through 0.45-µm nylon filters and agitated with a stirrer shaft rotating at 50 r.p.m. Cells were grown on nitrate-limited f/4 medium prepared with 0.2-µm filtered and autoclaved seawater (supplemented with Si in the case of *S. costatum*). We modified the nitrate concentration in the medium to obtain a molar N:P ratio of 10 and ensure N-limitation of growth. The concentrations of nitrate, phosphate and (for *S. costatum* only) silicate in the final medium were 181, 18 and 53 µmol L^−1^, respectively. Fresh medium was supplied to the culture vessels with high-precision peristaltic pumps (Watson Marlow 101 U/R). Another set of peristaltic pumps, integrated in the main bioreactor system and activated by a level sensor, controlled outflow rates to maintain a constant culture volume. Cultures were illuminated with a LED array delivering white light, under a 12:12 photoperiod, at a photon flux rate of 200 µmol m^−2^ s^−1^, which has been shown to be saturating for the growth of *S. costatum* [28], *E. huxleyi* [29] and *Synechococcus* [30].

We kept our cultures at a range of temperatures and dilution rates. The dilution rates used were 0.14, 0.35 and 0.60 d^−1^ for *S. costatum*; 0.09, 0.34 and 0.60 d^−1^ for *E. huxleyi*; and 0.10 and 0.30 d^−1^ for *Synechococcus*. These growth rates correspond to the range of phytoplankton growth rates commonly measured in open-ocean, oligotrophic regions [24, 31]. For each dilution rate, cultures were exposed to 4 different temperatures: 8, 12, 16 and 20 °C for *S. costatum*; 10, 14, 18 and 22°C for *E. huxleyi*; and 18, 22, 26 and 30 °C for *Synechococcus*. These temperature ranges were selected to avoid supraoptimal temperatures, based on previous studies on the thermal growth response of *S. costatum* [32], *E. huxleyi* [33] and *Synechococcus* [34]. All cultures were allowed to reach steady-state (constant biomass over time) and, for each combination of dilution rate and temperature, sampling for the determination of elemental composition and metabolic rates took place after an acclimation period of at least 10 days.

Standing stocks—We obtained cell counts of *S. costatum* and *E. huxleyi* under the microscope using Neubauer chambers. The abundance of *Synechococcus* was measured on fresh samples with a BD Accuri C6 flow cytometer. We determined chlorophyll *a* concentration fluorometrically on 5-mL samples filtered through GF/F filters and extracted with 90% acetone. The fluorescence signal was measured with a TD-700 Turner fluorometer calibrated with pure chlorophyll *a*. For the determination of particulate organic carbon (POC) and nitrogen (PON), duplicate 10-mL samples were filtered through pre-combusted GF/F filters, which were stored at −20 °C. For *E. huxleyi*, filters were exposed to concentrated HCl fumes to remove calcium carbonate. Before the analysis, filters were desiccated at room temperature for 48 h. Samples were analyzed with a Carlo Erba Instruments EA 1108 elemental analyzer (CE Instruments Ltd, Wigan, UK) using an acetanilide standard as a reference.

Metabolic rates—We measured photosynthetic CO_2_ fixation with the ^14^C-uptake technique, as described before [35]. Briefly, four 20-mL culture samples (three light and one dark samples) were amended with 5 µCi of NaH^14^CO_3_ and incubated for 2–3h under the same temperature and irradiance conditions experienced by the chemostat cultures. Experiments started 2 h after the beginning of the light phase of the photoperiod. After incubation, samples were filtered under low-vacuum pressure through 0.2-µm polycarbonate filters, which were then exposed overnight to concentrated HCl fumes to remove non-fixed inorganic ^14^C. After adding 5 mL of scintillation cocktail to each filter, sample radioactivity (DPM) was determined with a 1409–012 Wallac liquid scintillation counter. To compute hourly photosynthetic CO_2_ fixation rates, we subtracted the dark bottle DPM count from the light bottle DPM count and used a constant value of 2142 µmolC L^−1^ for the dissolved inorganic carbon content of seawater. Previous experiments conducted with cultures of 20 phytoplankton species [35] showed that C fixation rates obtained from short (2–3 h) incubations with ^14^C are strongly correlated with daily net POC increase measured in cultures (Fig. S1A in Supplementary Information). Short-term C fixation rates were also highly correlated with the C fixation rate measured during 12-h incubations (Fig. S1B). The amount of C fixed during 12 h (light phase of the photoperiod), *P*_12 h_, is calculated from the hourly C fixation rate in a short incubation, *P*_short_, using the equation:

3 $$P_{12\mathrm{h}} = - 19.5 + 9.5\,P_{\mathrm{short}}\,\left[ {r^2 = 0.94,p < 0.001,n = 16} \right]$$

Respiration was measured as the rate of dissolved O_2_ consumption in the dark. Five 30-mL borosilicate bottles were filled with culture. Two bottles were fixed immediately to determine the initial oxygen concentration, whereas the remaining three bottles were incubated for 5 h. Oxygen concentration was measured using the Winkler technique with a potentiometric endpoint. To obtain respiration rates in units of carbon, we applied a molar O_2_ consumption to CO_2_ production ratio of 1.4. Carbon-specific photosynthesis (P^C^) and respiration (R^C^) (units of h^−1^) were calculated by dividing hourly metabolic rates by POC concentration. To allow a direct comparison of the temperature- and nutrient-dependence of metabolic rates among the different species, we normalized P^C^ and R^C^ data by dividing them by the mean rates measured at a similar dilution rate (0.35 d^−1^ for *S. costatum*, 0.34 d^−1^ for *E. huxleyi* and 0.30 d^−1^ for *Synechococcus*). We calculated the daily respiration to photosynthesis ratio, R:P, by taking into account that respiration proceeds during all day (with the assumption that respiration is the same in the light and in the dark), whereas photosynthesis takes place only during the light phase:

4 $$R:P = (R_{\mathrm{short}} \times 24)/P_{12\mathrm{h}}$$

where R_short_ is the hourly rate of respiration measured during a 5-h incubation and *P*_12 h_ is calculated with Eq. 3.

Statistical analyses—We used ordinary least squares regression to calculate the slope of the linear relationship between 1/*kT* and the natural logarithm of mass-specific metabolic rates, which gives the *E*_a_. Throughout the study, when measurement error was present on both independent and dependent variables, we used reduced major axis regression to determine the parameters of the linear regression. The overall role of temperature and nutrient supply rate (dilution rate) as drivers of metabolic rates, as well as the existence of interactive effects, was assessed with multiple regression analysis on normalized data from all species combined, after standardizing the independent variables so that their effect sizes (coefficients in the linear regression model) could be comparable. We also applied multiple regression analysis separately to determine the effect of dilution rate (D) and temperature on carbon-specific photosynthesis in each individual species, according to the model:

5 $$ln\,{\mathrm{P}}^{\mathrm{C}} = c_{1} + c_{2}ln\,D - E_{\mathrm{a}}({1/kT})$$

We used the data compiled by [18] to calculate average values of *E*_a_ for growth rate in nutrient-replete cultures of *S. costatum*, *E. huxleyi* and *Synechococcus*. *E*_a_ values in [18] were computed only with data from the growing part of the temperature response curve, so the relevant temperature ranges were similar to the ones used in our experiments. Also in agreement with our experiments, growth irradiance in the original studies was saturating. *E*_*a*_ values in nutrient-limited versus nutrient-replete cultures were compared using the Mann–Whitney *U*-test (two-tailed). All statistical analyses were carried out with SPSS Statistics v. 24.

## Results

### Effect of nutrient-limited growth on cellular composition and carbon fixation

**Fig. 1:**
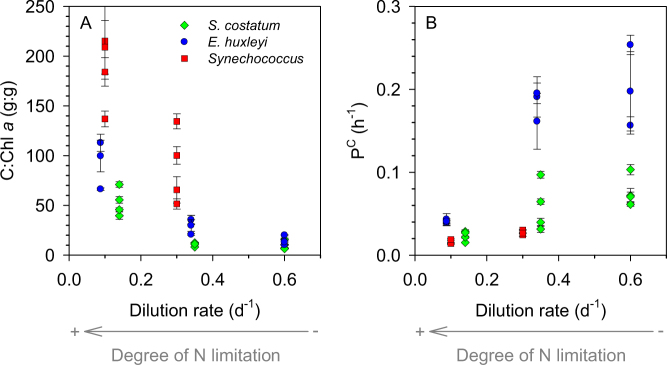
Relationship between dilution rate and (**a**) carbon to chlorophyll *a* ratio (C:Chl *a*) and (**b**) carbon-specific photosynthetic rate (P^C^) in *Skeletonema costatum*, *Emiliania huxleyi* and *Synechococcus* growing under N-limited conditions in continuous cultures. The different datapoints at each dilution rate correspond to different growth temperatures. Bars indicate standard deviation

The different dilution rates in our chemostats provided a range of degrees of nitrogen limitation that reflected upon the biochemical composition and metabolic rate of the populations. The carbon to chlorophyll *a* ratio (C:Chl *a*) increased as dilution rates became slower (Fig. 1a). In *S. costatum* and *E. huxleyi*, C:Chl *a* increased from 10–20 gC gChl^−1^ at the fastest dilution rate (0.6 d^−1^) to 40–120 gC gChl^−1^ at the slowest dilution rate (ca. 0.1 d^−1^). C:Chl *a* in *Synechococcus* took values in the range 50–130 gC gChl^−1^ at 0.3 d^−1^ and increased to 130–220 gC gChl^−1^ at 0.1 d^−1^. The log-log relationship between dilution rate and C:Chl *a*, for all species combined, had a slope of −1.5 (Fig. S2). The effect of nutrient supply (dilution rate) on C:Chl *a* resulted from changes in the cellular content of both C and Chl *a*, although the latter showed higher variability (Fig. S3). Cells growing at the slowest dilution rate had more C per cell than those growing at faster dilution rates, whereas the cellular Chl *a* content tended to increase with dilution rate (Fig. S3). Cell carbon decreased or remained largely unchanged as temperature increased (Fig. S4). Growth temperature had a marked impact on C:Chl *a* (Fig. S5). In most combinations of species and dilution rate, C:Chl *a* increased by a factor of 1.5 to 2 from the warmest to the coldest temperature. The C:N elemental ratio of particulate organic matter responded to the degree of nutrient limitation, taking lower values at faster dilution rates in both *S. costatum* and *E. huxleyi* (Fig. S6B,D). With all data pooled together, the rate of nutrient supply had a significant effect on C:N, whereas temperature had no significant effect (Table S1).

Carbon-specific carbon fixation rate (*P*^C^), equivalent to the biomass turnover rate, increased with increasing dilution rate (Fig. 1b) (Pearson’s *r* = 0.61, *p* = 0.0004, *n* = 29). Due to the opposite patterns of variability of C:Chl *a* and P^C^ as a function of dilution rate, there was no correlation between Chl-specific photosynthesis and dilution rate (Pearson’s *r* = −0.24, *p* > 0.2, *n* = 28).

### Role of temperature and nutrient limitation in the control of metabolic rates

**Fig. 2:**
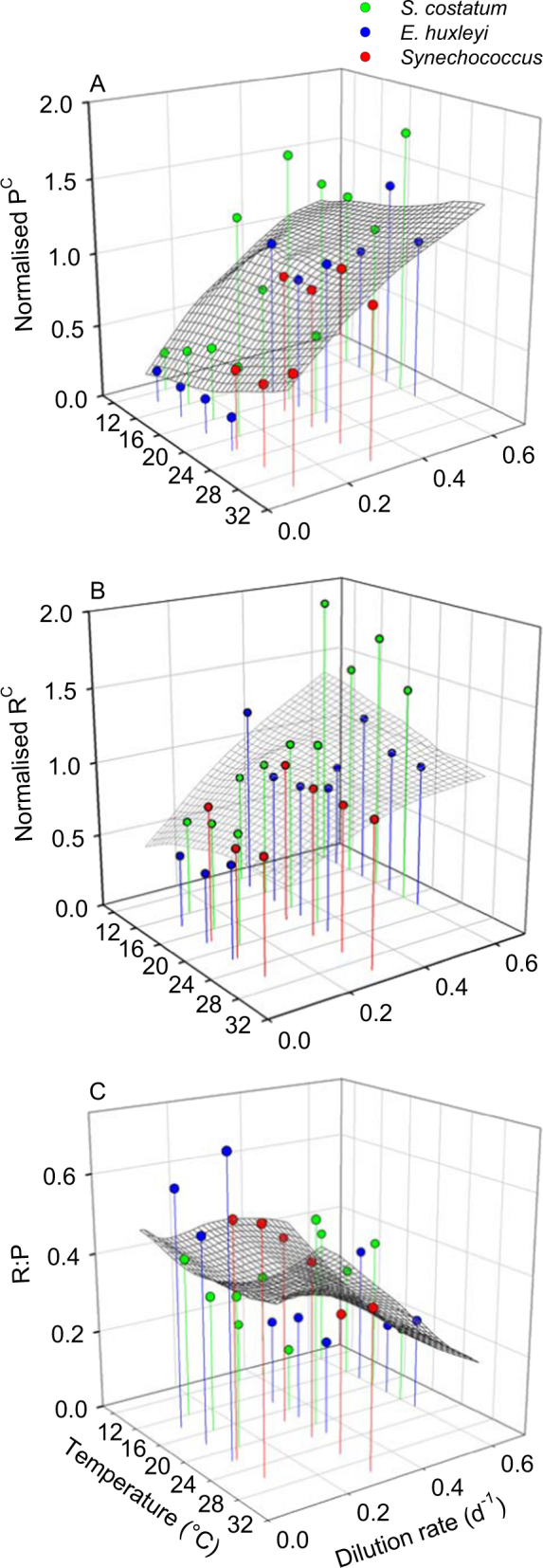
Normalized P^C^ and R^C^, and respiration to photosynthesis ratio (R:P) in (**a**) *S. costatum*, (**b**) *E. huxleyi* and (**c**) *Synechococcus* under different temperatures and growth rates. For each species, P^C^ and R^C^ data were normalized by dividing them by the mean rate measured at a dilution rate of 0.35 d^−1^ (*S. costatum*), 0.34 d^−1^ (*E. huxleyi*) and 0.30 d^−1^ (*Synechococcus*). The smoothed surface was obtained with local regression (LOESS) using tricube weighting and a polynomial of degree 2

Both P^C^ and R^C^ increased markedly with dilution rate and were largely independent of temperature in all species (Fig. S7). When normalized P^C^ and R^C^ values are plotted against temperature and dilution rate for all species together (Fig. 2), the pattern of nutrient supply-dependent and temperature-independent metabolic rates is evident. Multiple regression analysis confirmed a highly significant effect of nutrient supply rate (dilution rate) upon both P^C^ and R^C^, with *R*^2^ values > 0.5, whereas temperature had no significant effect (Table S1). We observed a strong correlation between P^C^ and R^C^ (Pearson’s *r* = 0.90, *n* = 27, *p* < 0.0001; Fig. S8A) and consequently the variability in the respiration to photosynthesis ratio (R:P), which took a mean value of 0.37 (95% CI = 0.31, 0.43), was smaller than that of P^C^ and R^C^ (Fig. 2c). However, photosynthetic carbon fixation increased with dilution rate faster than respiration did (Fig. 2a, b) and as a result R:P showed a moderate but significant increase with decreasing dilution rate, whereas it remained invariant with respect to temperature (Fig. 2c, Table S1). The interaction between temperature and dilution rate was not significant for P^C^ and R^C^ but was marginally significant for R:P (Table S1). The intercept of the linear relationship between growth rate and R^C^ can be used to calculate µ_0_, the basal metabolic rate, which took a value of 0.046 (SE = 0.017), 0.163 (SE = 0.051), and 0.072 (SE = 0.006) d^−1^ for *S. costatum*, *E. huxleyi* and *Synechococcus*, respectively (Fig. S8B).

The effect of dilution rate on metabolic rates was also evident when cell-specific rates were examined (Fig. S9). Despite the fact that cell carbon tended to decrease or remain unchanged as dilution rate increased (Fig. S3), cell-specific rates of photosynthetic C fixation increased with dilution rate (Fig. S9A,C,E). In contrast, cell-specific rates of respiration remained largely invariant with respect to nutrient supply (Fig. S9B,D,F).

The multiple regression analysis performed separately on each species confirmed the strong effect of dilution rate, and the non-significant effect of temperature, on photosynthetic C fixation (Table S2). The intercept of the temperature and dilution rate-dependent model was not significantly different among species, indicating that, for the same temperature and nutrient supply rate, all species sustained broadly similar rates of biomass-specific C fixation.

**Fig. 3:**
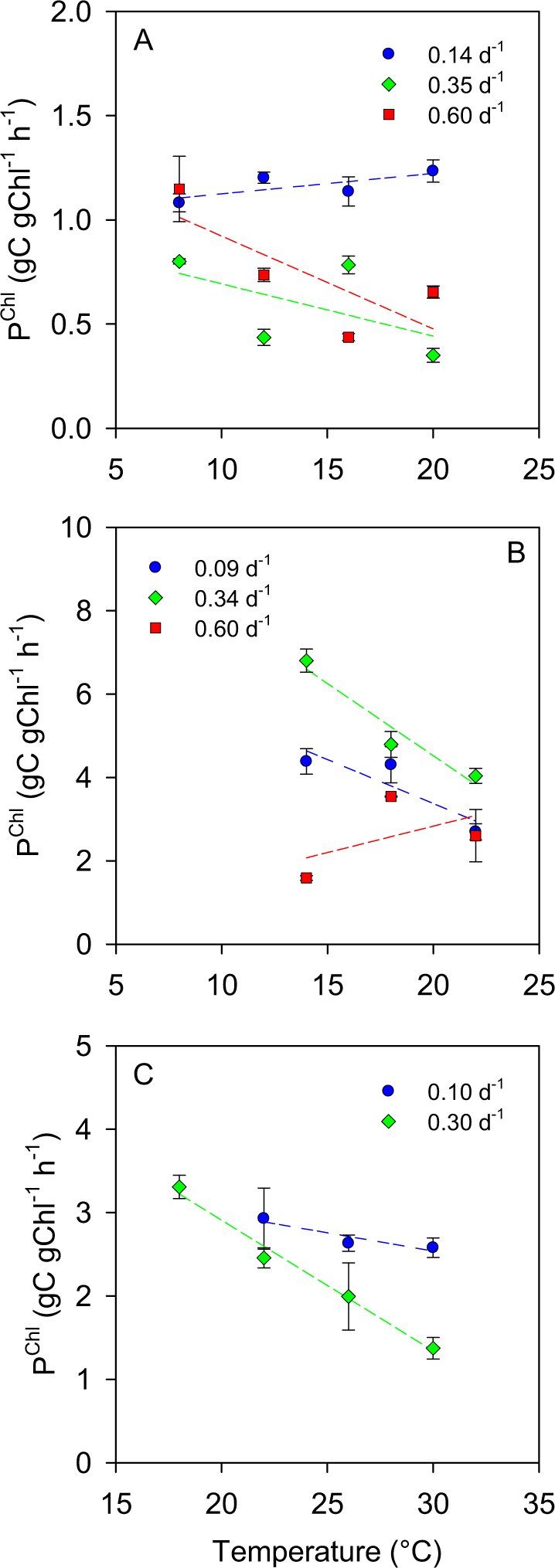
Temperature dependence of chlorophyll *a*-specific C fixation rate (P^Chl^) in (**a**) *S. costatum*, (**b**) *E. huxleyi* and (**c**) *Synechococcus* under N-limited continuous growth at different dilution rates. Bars indicate standard deviation and dashed lines are the ordinary least squares regression fits

Given that the cellular content of Chl *a* was dependent on both nutrient supply (Fig. S3) and temperature (Fig. S5), we investigated also the variability in Chl-specific photosynthesis. In all species, C fixation per unit Chl *a* tended to decrease or remain unchanged with increasing temperature (Fig. 3).

### Activation energy of metabolic rates

**Fig. 4:**
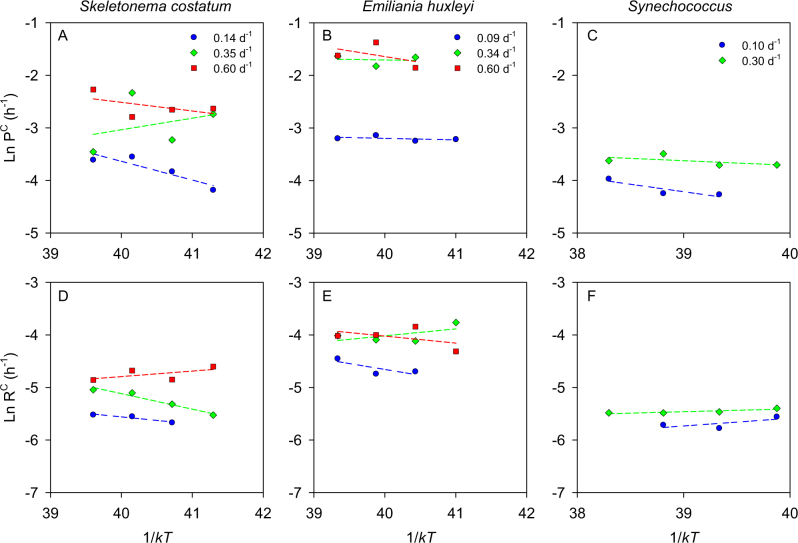
Arrhenius plots for carbon-specific photosynthesis (P^C^) and respiration (R^C^) in *S. costatum* (left), *E. huxleyi* (middle) and *Synechococcus* (right) growing in N-limited chemostats at different dilution rates. Dashed lines represent the linear fit (OLS regression) between inverse temperature (1/*kT*) and the carbon-specific metabolic rate. Slope values are given in Table S3

**Fig. 5:**
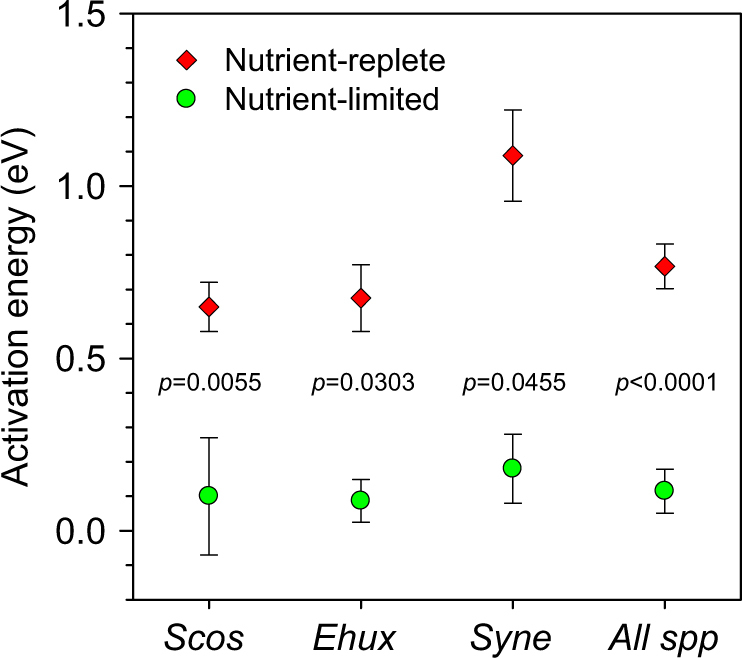
Comparison between the activation energy (*E*_a_) of growth rate under nutrient-replete growth conditions in batch cultures and the *E*_*a*_ of carbon-specific photosynthesis measured under nutrient-limited growth in chemostat cultures of *S. costatum* (*n* = 3), *E. huxleyi* (*n* = 3) and *Synechococcus* (*n* = 2). Mean *E*_a_ values for *S. costatum* (*n* = 11), *E. huxleyi* (*n* = 7) and *Synechococcus* (*n* = 6) under nutrient-replete conditions were calculated from the data compilation in [18]. The significance values (two-tailed) correspond to the Mann–Whitney *U*-test carried out to compare the *E*_a_ values between nutrient-replete and nutrient-limited conditions. Bars indicate the SE

The Arrhenius plots indicated that, with a few exceptions, both photosynthesis and respiration increase with temperature at a much slower pace than predicted by the MTE (Fig. 4, Table S3). Out of 16 determinations of *E*_a_, only in one case (respiration in *S.costatum* at 0.35 d^−1^) did the obtained estimate differ significantly from 0. For the ensemble of all species and dilution rates, *E*_a_ took a mean ( ± SE) value of 0.11 ± 0.06 eV for photosynthesis (*n* = 8) and 0.04 ± 0.17 eV for respiration (*n* = 8). Considering all metabolic rate measurements pooled together, the mean *E*_a_ was 0.08 ± 0.04 eV (*n* = 16). The *E*_a_ values for photosynthesis measured under nutrient-limited conditions were significantly lower (Mann–Whitney *U*-test) than those reported in the literature [18] for nutrient-replete cultures of the same species (Fig. 5). The mean *E*_a_ of growth rate was 0.65 ± 0.07 (*n* = 11), 0.68 ± 0.09 (*n* = 7) and 1.09 ± 0.13 (*n* = 6) eV for nutrient-replete *S. costatum*, *E. huxleyi* and *Synechococcus*, respectively, while the corresponding *E*_a_ values for photosynthesis under nutrient limitation in our populations were 0.10 ± 0.17 (*n* = 3), 0.09 ± 0.06 (*n* = 3), and 0.18 ± 0.07 (*n* = 2) eV.

## Discussion

We conducted our measurements on three species that differ widely in phylogenetic affiliation, functional traits and ecological niche. Yet, the observed relationships between temperature, nutrient supply and both biochemical composition and metabolic rate depicted a coherent pattern valid for all species. The carbon to chlorophyll *a* ratio (C:Chl) tended to increase with decreasing dilution rate, as has often been observed in nutrient-limited phytoplankton cultures [36–38]. This pattern arises because a growing degree of nitrogen limitation (slower dilution rates) leads to a reduction in the size of the nitrogen-rich, light-harvesting apparatus and hence a decreased chlorophyll content [39]. The fact that C:Chl increases with decreasing nutrient-limited growth rate means that Chl-normalized C fixation rate gives a biased estimate of biomass turnover rate, and can be misleading when testing hypotheses that concern the relationship between environmental drivers and phytoplankton metabolism and growth [40]. Indeed, we found no correlation between Chl-specific photosynthesis and nutrient supply rate in our study, which illustrates the fact that phytoplankton respond to nutrient limitation by changing the number and size, but not the efficiency, of photosystems [41].

The C:N elemental ratio of marine phytoplankton has a major impact on both the transfer of energy through food webs and the efficiency of the biological pump to export organic carbon towards deep waters. As reported in previous studies [42], we found a significant effect of nutrient supply on elemental composition, such that stronger nitrogen limitation led to an increased C:N ratio in both *S. costatum* and *E. huxleyi*. In contrast, temperature had no effect upon C:N, which supports the view that nutrient availability alone can explain most of the variability in phytoplankton elemental stoichiometry [43].

Temperature-dependent changes in the allocation of resources into photosynthetic machinery can affect the relationship between temperature and both individual and biomass-specific C fixation rates. The MTE predicts that C fixation per unit photosynthetic complex increases with temperature [44]. However, in our nutrient-limited cultures, we found that Chl-specific C fixation tended to decrease or remain unchanged with increasing temperature. Thus, in spite of having a higher Chl *a* content, cells under warmer temperatures sustained similar biomass-specific photosynthetic rates than those growing under colder temperatures.

Temperature-related changes in cell size could also potentially have an impact on the response of metabolic rates to rising temperature. Warmer temperature induces smaller cell size in protists [45], a pattern we observed in some of our experiments. Hence, according to the MTE, which predicts a faster pace of metabolism with decreasing body size [3], one would have expected higher metabolic rates at warmer temperatures. Yet, biomass-specific photosynthesis and respiration remained largely unaffected by temperature in our N-limited populations. Earlier studies have shown that individual metabolic rates in phytoplankton do not follow the ¾ power size scaling commonly observed in multicellular organisms but instead scale isometrically with cell size [23, 46]. This means that, as a first-order approximation, phytoplankton cells of all sizes sustain broadly comparable maximum rates of biomass-specific metabolism. Accordingly, we found that, when compared at the same temperature and nutrient supply rate, all three species had similar rates of photosynthesis per unit biomass.

Carbon-specific carbon fixation rate (P^C^) increased markedly with increasing dilution rate in all species, reflecting the coupling between population growth rate and biomass turnover rate, which arises from the fact that reproduction is ultimately fueled by metabolism [3]. A similar covariation between growth rates and P^C^ has been observed before in nutrient-limited chemostat cultures growing under different dilution rates [36, 47] and in a study of the size dependence of phytoplankton metabolism and growth in batch cultures [35].

We found that respiration tended to increase with dilution rate at a slower pace than photosynthesis did, which resulted in enhanced R:P values at the slowest dilution rates. These results agree with previous observations showing that respiratory losses tend to become a larger fraction of photosynthetic carbon fixation under suboptimal conditions [46, 48, 49]. Such a pattern arises from the existence of basal metabolic maintenance costs that are largely independent of biosynthesis rates. The basal metabolic rates we calculated (0.05, 0.16 and 0.07 d^−1^ for *S. costatum*, *E. huxleyi* and *Synechococcus*, respectively) agree well with earlier estimates [48], which gave a mean value of 0.08 d^−1^ (95% CI = 0.04, 0.13) in nutrient-limited cultures of phytoplankton species from various taxa.

R:P is a key variable that determines the efficiency of carbon use by primary producers (the fraction of fixed carbon available for allocation to growth) and their net contribution to the carbon cycle [50]. Given that in photoautotrophs respiration is ultimately constrained by CO_2_ fixation [44], the temperature dependences of phytoplankton respiration and photosynthesis are similar [51]. However, experimental studies have found that, over temporal scales of a few generations and under resource-replete conditions, microalgal respiration can be more responsive to temperature than photosynthesis [50, 52], such that warmer temperatures lead to increased R:P. In contrast, our data show that temperature has no effect on the R:P of fully-acclimated phytoplankton under nutrient-limited, continuous growth. Overall, our results suggest that nutrient supply has a larger role than temperature in controlling the efficiency of photosynthetic carbon conversion into new biomass.

Our determinations of activation energy (*E*_a_) of photosynthesis and respiration show that the temperature dependence of phytoplankton metabolic rates is suppressed by nutrient limitation. The mean values of *E*_a_ observed in our nutrient-limited chemostats were much lower than those determined for the growth rate of cultures of the same species growing under nutrient-replete conditions [18]. They were are also lower than the value of 0.32 eV calculated for C_3_ terrestrial plants [44]. The lack of temperature dependence of metabolic rates under nutrient limitation is comparable with the observation that strong light limitation greatly diminishes the temperature sensitivity of phytoplankton growth [53]. While our experiments were conducted under saturating light levels, additional studies are needed to address the interactive effect of temperature and nutrient supply in light-limited conditions.

Metabolic acclimation to temperature in photosynthetic unicells can involve changes in both the abundance and the specific activity of catalysts [54]. Biomass-specific carbon fixation rates can be maintained under cold temperatures if the abundance of Rubisco increases sufficiently to compensate for the cold-induced reduction in its substrate turnover rate. For instance, polar diatoms have a relative Rubisco content 10 times higher than diatoms growing at warm temperatures [55]. However, this strategy heavily increases the cellular demands for nitrogen and thus is unlikely to be used by strongly N-limited cells.

An alternative explanation for the lack of temperature dependence of metabolic rates in nutrient-limited populations can be found in enzyme kinetics. Under conditions of nutrient limitation, intracellular substrate abundance decreases and therefore the temperature dependence of enzyme half-saturation constant (*k*_m_) becomes more relevant than that of the maximum reaction rate (*V*_*m*_) [4, 56]. Increasing temperature leads to higher kinetic energy of reactants and increased rates of collision, as well as higher structural flexibility of enzymes, all of which promote faster catalytic rates [5, 57]. However, higher structural flexibility also results in active sites with a reduced ability for ligand recognition and binding and lower kinetic efficiency, which results in lower affinity (higher *k*_m_). The *k*_m_ of most enzymes increases with temperature, with Q_10_ values similar to, or higher than, those of *V*_m_ [4, 57]. In phytoplankton, the *k*_m_ of Rubisco has a Q_10_ of approximately 2 [55], and the *k*_m_ of nitrate uptake and growth in nutrient-limited cultures has a Q_10_ higher than 2 [58, 59], while the Q_10_ of *V*_m_ in a wide range of enzymes involved in both anabolic and catabolic pathways takes a mean value of 2.1 ± 0.4 [54]. If both *V*_m_ and *k*_m_ have the same temperature sensitivity, the realized reaction rates at low substrate concentration ([S] < *k*_m_) can be similar at divergent temperatures, and the resulting temperature dependence of metabolic rate becomes very small (Fig. S10). This mechanism, whose importance is well recognized in terrestrial ecology to explain thermal adaptation of organic matter decomposition in soils [4, 60, 61], could also explain the lack of temperature sensitivity of phytoplankton metabolic rates under conditions of nutrient limitation.

We have provided the first experimental determinations of the activation energy of metabolic rate in phytoplankton experiencing chronic nutrient limitation of growth. Our results stress the need to consider resource limitation when using MTE-based approaches to understand the environmental control of metabolic activity and, in addition, have implications for the prediction of climate change impacts on ocean biogeochemistry. Modeling studies have suggested that warming can have a direct, stimulating effect on ocean net primary production, particularly in low-latitude regions [12, 13]. This increased productivity would be associated with a faster nutrient recycling through the microbial loop and an increase in regenerated production. Our results, however, suggest that this effect is unlikely to occur in ocean regions where phytoplankton growth is severely limited by nutrient availability, such as the nitrogen-limited subtropical gyres or the iron-limited high-nutrient, low-chlorophyll regions [25]. Hence, it can be expected that direct responses of primary production to warming will vary widely among regions. The stimulating effect of increasing temperatures on phytoplankton production and growth may be significant in coastal and upwelling regions [62], but is likely to be minor in oligotrophic waters. Previous studies on the interaction between temperature and resources in aquatic ecosystems have emphasized the role of temperature in regulating the effect of nutrient supply upon metabolic rates [1]. In contrast, our results suggest that nutrient availability controls the temperature dependence of metabolism, such that the direct effect of increasing temperature on metabolic rates is virtually absent under nutrient-limiting conditions. Furthermore, we have shown that nutrient supply explains most of the variability in the photosynthesis and respiration of phytoplankton, whereas temperature plays a much smaller role. Indirect effects of temperature upon resource supply are therefore likely to dominate the response of phytoplankton growth and productivity to ocean warming.

## Electronic supplementary material

Supplementary information

Dataset 1
