# Supplementary material for: Nutrient limitation suppresses the temperature dependence of phytoplankton metabolic rates
Source: ISME J. 2018 Apr 25;12(7):1836–45. doi: 10.1038/s41396-018-0105-1 (PMC6018665; doi:10.1038/s41396-018-0105-1)
Supplement: Supplementary file 1 — Supplementary information [file 41396_2018_105_MOESM1_ESM.pdf]

Supplementary Information

Nutrient limitation suppresses the temperature dependence of phytoplankton metabolic rates

E. Marañón[1] (em@uvigo.es), M. P. Lorenzo[1], P. Cermeño[2], B. Mouriño-Carballido[1]

[1]Departamento de Ecología y Biología Animal, Universidade de Vigo, 36310 Vigo, Spain

[2]Instituto de Ciencias del Mar, CSIC, Passeig Maritim de la Barceloneta 37-49, 08003 Barcelona, Spain

Table S1. Multiple regression analysis with temperature (Temp, °C) and dilution rate (D, d^{-1}) as independent variables and normalized carbon-specific photosynthesis (nP^C, h^{-1}), normalized carbon-specific respiration (nR^C, h^{-1}), the respiration to photosynthesis ratio (R:P) and the molar carbon to nitrogen ratio (C:N) as dependent variables. The analysis was conducted with data from all species pooled together. For each species, P^C and R^C values were normalized by dividing them by the mean value measured at a dilution rate of 0.35 d^{-1} (*S. costatum*), 0.34 d^{-1} (*E. huxleyi*) and 0.30 d^{-1} (*Synechococcus*). To ensure that the regression coefficients for each independent variable (effect sizes) are comparable, temperature and dilution rate values were standardized by calculating the difference with the mean and then dividing by the standard deviation. The multiple regression model is $y = b_0 + b_1 x_1 + b_2 x_2 + b_3 x_1 x_2$ where y is the metabolic rate, x_1 and x_2 are temperature and dilution rate, respectively, and b_0, b_1, b_2 and b_3 are the coefficients. 95% confidence intervals for the standardized coefficient estimates are given in parentheses. Also shown are the coefficient of determination of the multiple regression model (R^2), the ANOVA F and p values, and the number of observations.

Dependent variable	Temp. b_1	p	D b_2	p	Temp. × D b_3	p	R^2	F	p	n
nP^C	0.094 (-0.014, 0.203)	0.08	0.355 (0.247-0.462)	<0.0001	-0.005 (-0.124, 0.113)	0.92	0.66	16.4	<0.0001	29
nRC	0.012 (-0.086, 0.110)	0.80	0.236 (0.137, 0.335)	<0.0001	-0.065 (-0.179, 0.049)	0.25	0.54	9.8	0.0002	29
R:P	0.023 (-0.023, 0.071)	0.31	-0.098 (-0.146, -0.049)	0.0003	-0.060 (-0.115, -0.005)	0.04	0.54	8.5	0.0006	26
C:N	-0.271 (-1.331, 0.789)	0.61	-1.815 (-2.870, -0.761)	0.0014	0.197 (-0.959, 1.355)	0.73	0.33	4.5	0.0105	32

Table S2. Multiple regression analysis with natural logarithm of carbon-specific photosynthesis ($\ln P^C$, h^{-1}) as dependent variable and inverse temperature (1/*kT*, °K) and natural logarithm of dilution rate (lnD, d^{-1}) as independent variables. The analysis was conducted separately for *S. costatum* (*Scos*), *E. huxleyi* (*Ehux*) and *Synechococcus* (*Syne*). The multiple regression model is $\ln P^C = c_1 + c_2 \ln D - E_a (1/kT)$. 95% confidence intervals for the standardized coefficient estimates are given in parentheses. Also shown are the coefficient of determination of the multiple regression model (R^2), the ANOVA F and *p* values, and the number of observations.

	c_1	*p*	c_2	*p*	$-E_a$	*p*	R^2	F	*p*	*n*
Scos	1.94 (-12.90, 16.79)	0.77	0.84 (0.45-1.22)	<0.001	-0.10 (-0.47, 0.26)	0.55	0.73	12.4	0.003	12
Ehux	2.71 (-16.1, 21.5)	0.74	0.87 (0.61, 1.13)	<0.001	-0.09 (-0.56, 0.37)	0.65	0.92	33.4	<0.001	9
Syne	2.55 (-5.9, 11.0)	0.45	0.52 (0.30, 0.73)	0.003	-0.14 (-0.36, 0.07)	0.14	0.92	22.7	0.007	7

Table S3. Slope ($-E_a$, eV) of the ordinary least squares linear regression between $1/kT$ and the natural logarithm of carbon-specific photosynthesis (P^C) and respiration (R^C) for each species under each dilution rate (D). The 95% confidence interval of $-E_a$ and the *p*-value are also given.

	D, d^{-1}	$-E_a$	95% CI	*p*
P^C				
Skeletonema costatum	0.14	-0.36	-0.86, 0.15	0.09
	0.35	0.22	-1.77, 2.21	0.68
	0.60	-0.16	-0.94, 0.62	0.46
Emiliania huxleyi	0.09	-0.03	-0.20, 0.14	0.54
	0.34	-0.02	-2.42, 2.38	0.93
	0.60	-0.21	-0.56, 0.67	0.67
Synechococcus	0.10	-0.28	-2.10, 1.54	0.29
	0.30	-0.08	-0.44, 0.27	0.44
R^C				
Skeletonema costatum	0.14	-0.13	-0.67, 0.40	0.19
	0.35	-0.29	-0.48, -0.10	0.02
	0.60	0.10	-0.32, 0.53	0.39
Emiliania huxleyi	0.09	-0.22	-2.49, 2.04	0.43
	0.34	0.14	-0.41, 0.68	0.41
	0.60	-0.13	-0.86, 0.59	0.51
Synechococcus	0.10	0.15	-1.77, 2.07	0.51
	0.30	0.05	-0.03, 0.14	0.12

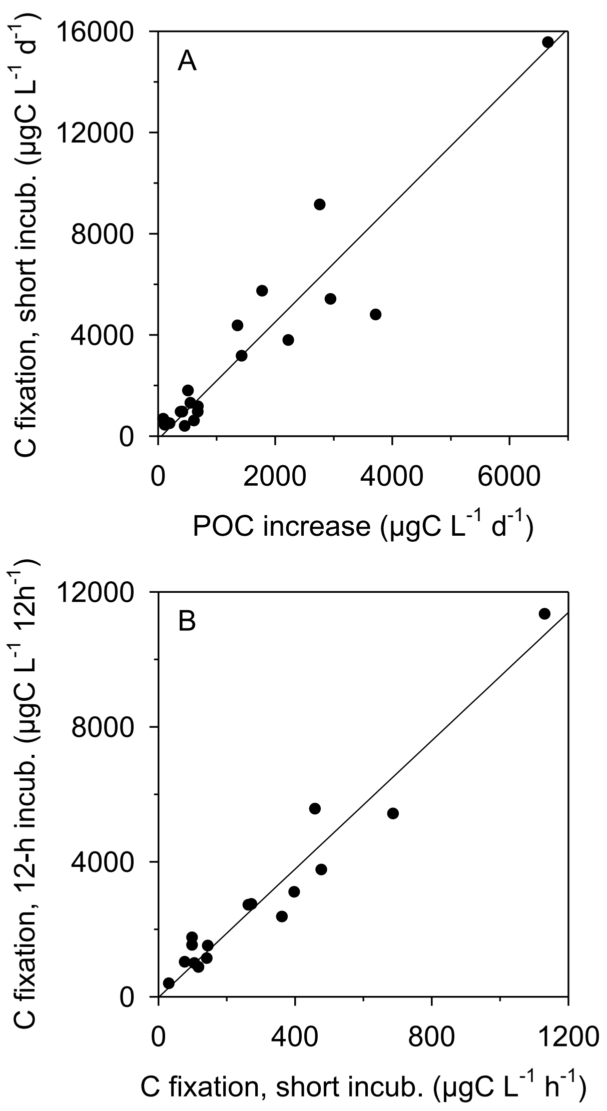

Fig. S1. A) Relationship between the daily increase in particulate organic carbon (POC) and the daily rate of C fixation estimated from short (2-3 h) ^{14}C incubations in batch cultures of 20 phytoplankton species including *S. costatum*, *E. huxleyi* and *Synechococcus*. Each datapoint corresponds to a different species. The daily C fixation rate was calculated by multiplying the hourly rate, measured during a short incubation, by 12 h (the duration of the light phase of the photoperiod). The reduced major axis (r. m. a.) regression line is $y = -131.5 + 2.3\,x$ ($r^2 = 0.88$, $p < 0.001$, $n = 20$). B) Relationship between the hourly rate of C fixation in short ^{14}C incubations and C fixation measured during 12-h incubations. The r. m. a. regression line is $y = -19.5 + 9.5\,x$ ($r^2 = 0.94$, $p < 0.001$, $n = 16$).

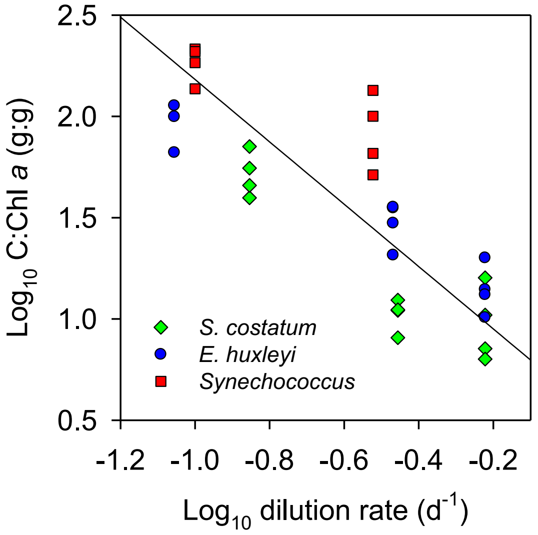

Fig. S2. Log-log relationship between dilution rate and the carbon to chlorophyll *a* ratio in *S. costatum*, *E. huxleyi* and *Synechococcus*. The OLS regression fit for all data combined is $y = 0.64 - 1.54\,x$ ($r^2 = 0.68$, $p < 0.001$, $n = 31$).

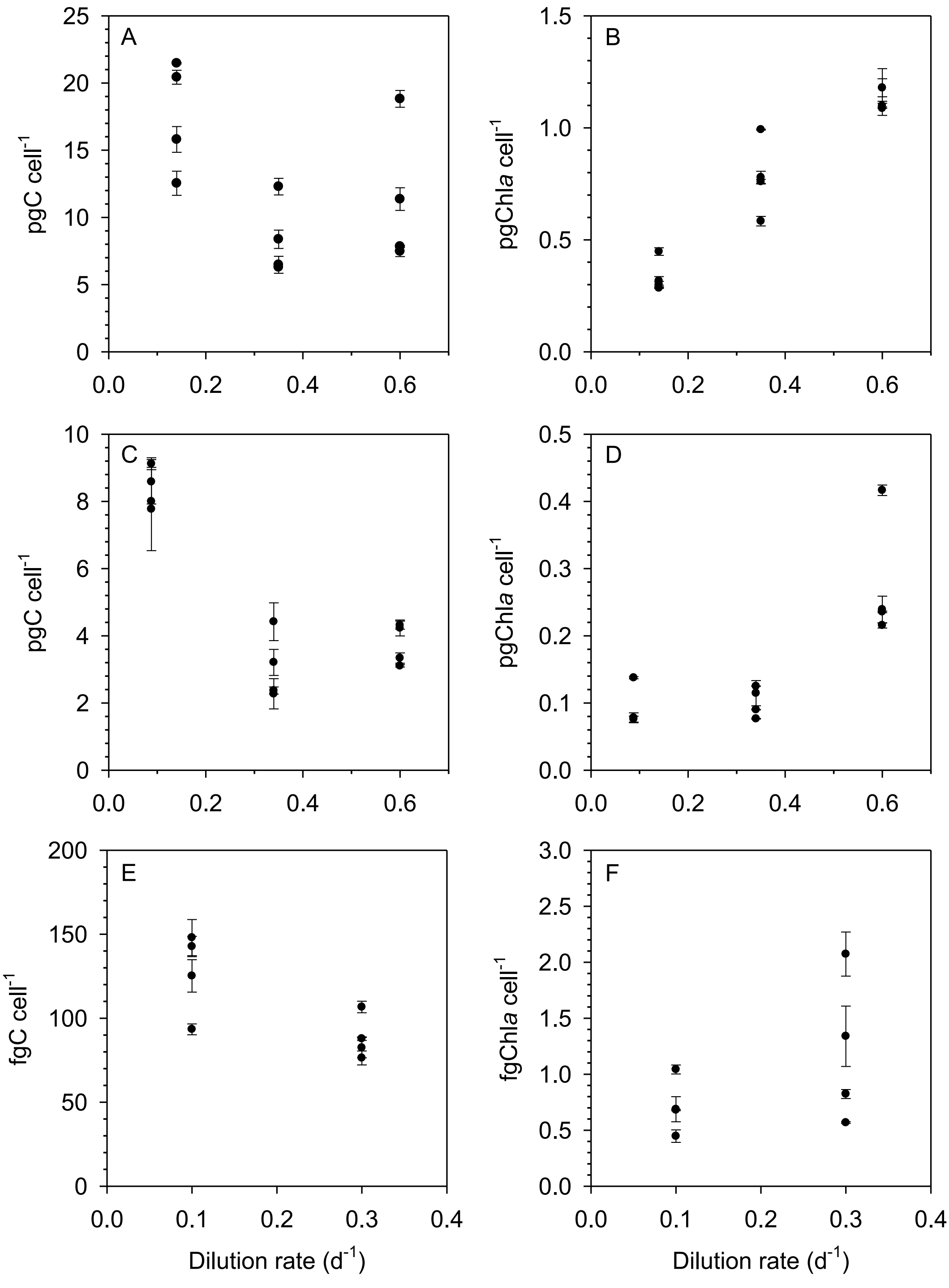

Fig. S3. Relationship between dilution rate and the cellular content of (left) carbon and (right) chlorophyll *a* in A,B) *S. costatum*, C,D) *E. huxleyi* and E,F) *Synechococcus*. Bars indicate standard deviation.

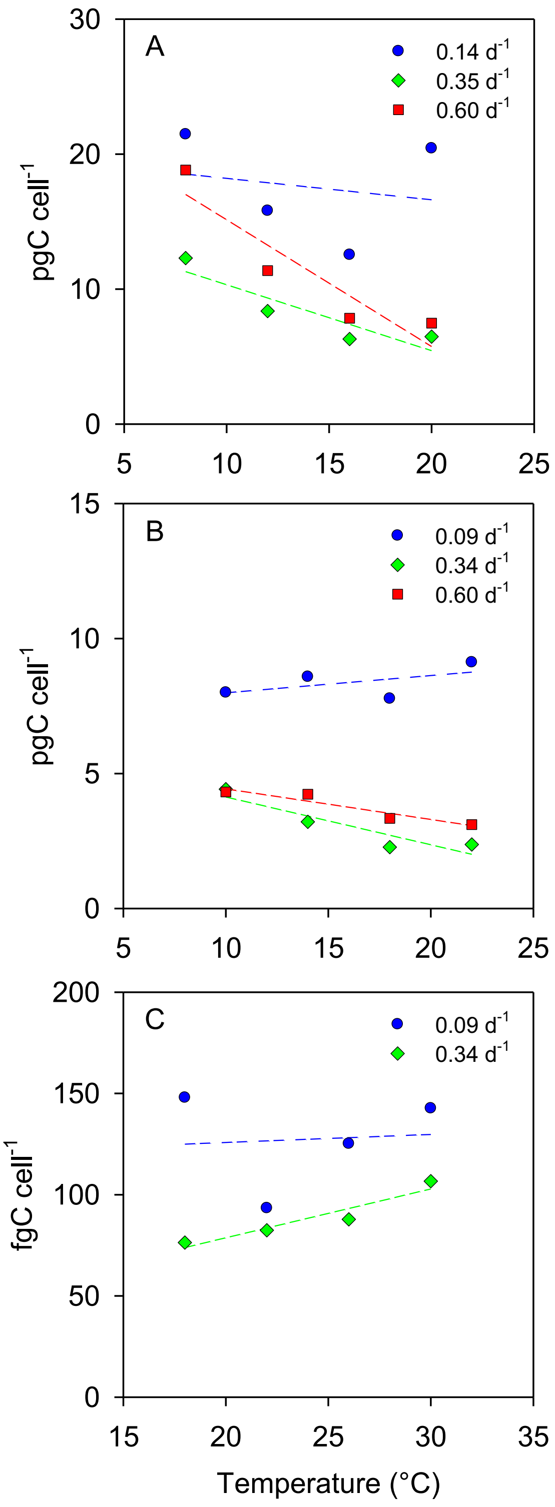

Fig. S4. Relationship between temperature and cellular carbon in N-limited chemostats of A) *S. costatum*, B) *E. huxleyi* and C) *Synechococcus* at different dilution rates. Lines are the OLS regression fits.

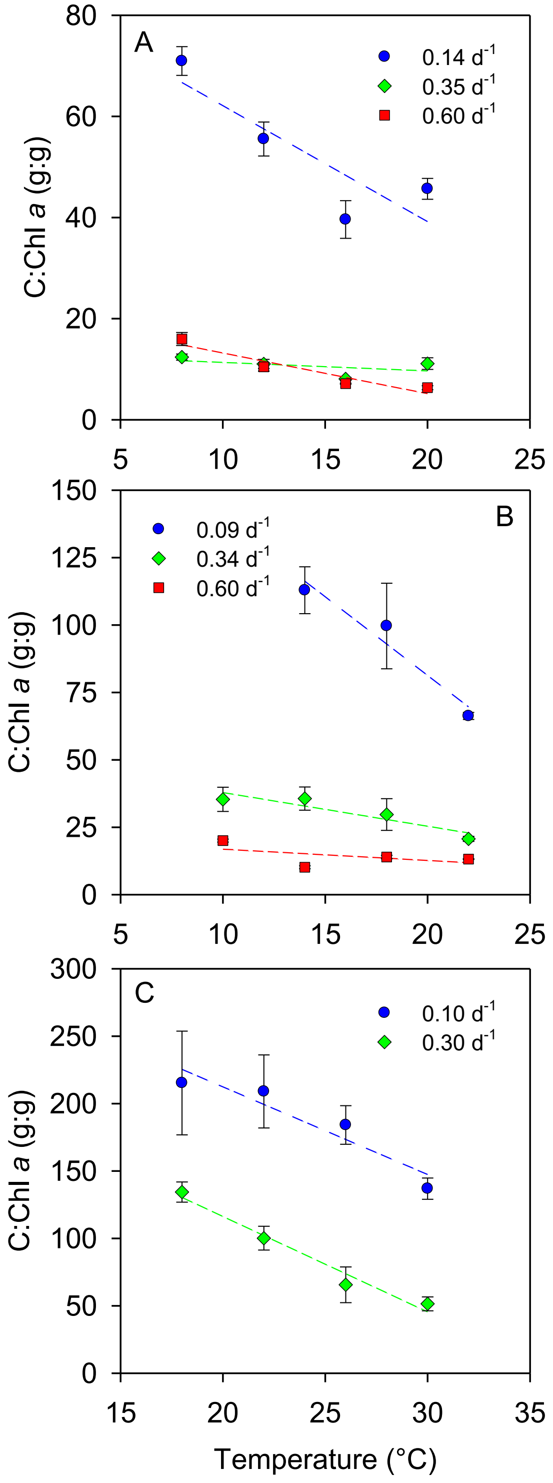

Fig. S5. Temperature dependence of the carbon to chlorophyll *a* ratio (C:Chl *a*) in A) *S. costatum*, B) *E. huxleyi* and C) *Synechococcus* under N-limited continuous growth at different dilution rates. Bars indicate standard deviation and dashed lines are the OLS regression fits.

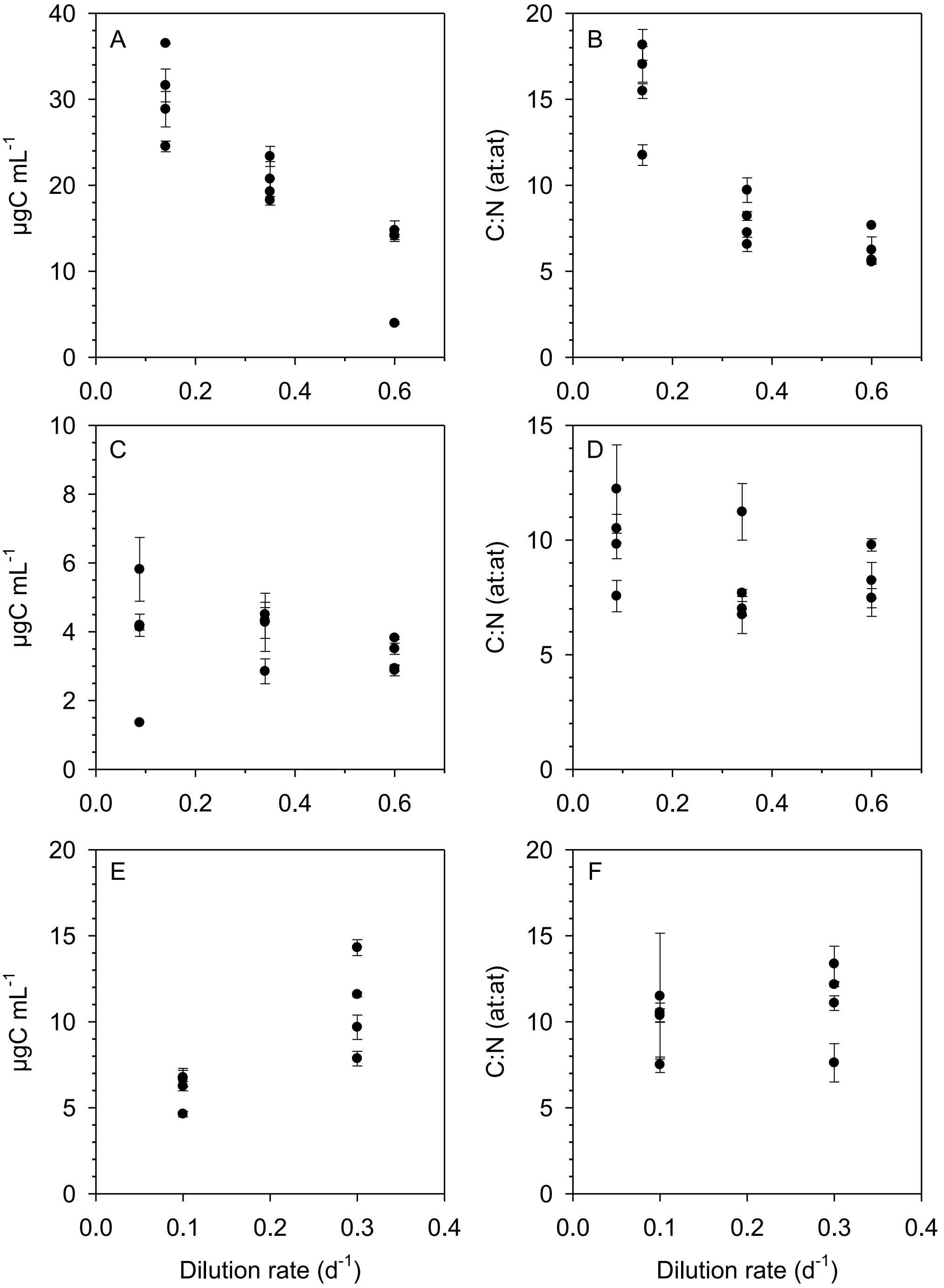

Fig. S6. Relationship between dilution rate and (left) the concentration of particulate organic carbon and (right) the C:N ratio of suspended organic matter in A,B) *S. costatum*, C,D) *E. huxleyi* and E,F) *Synechococcus*. Bars indicate standard deviation.

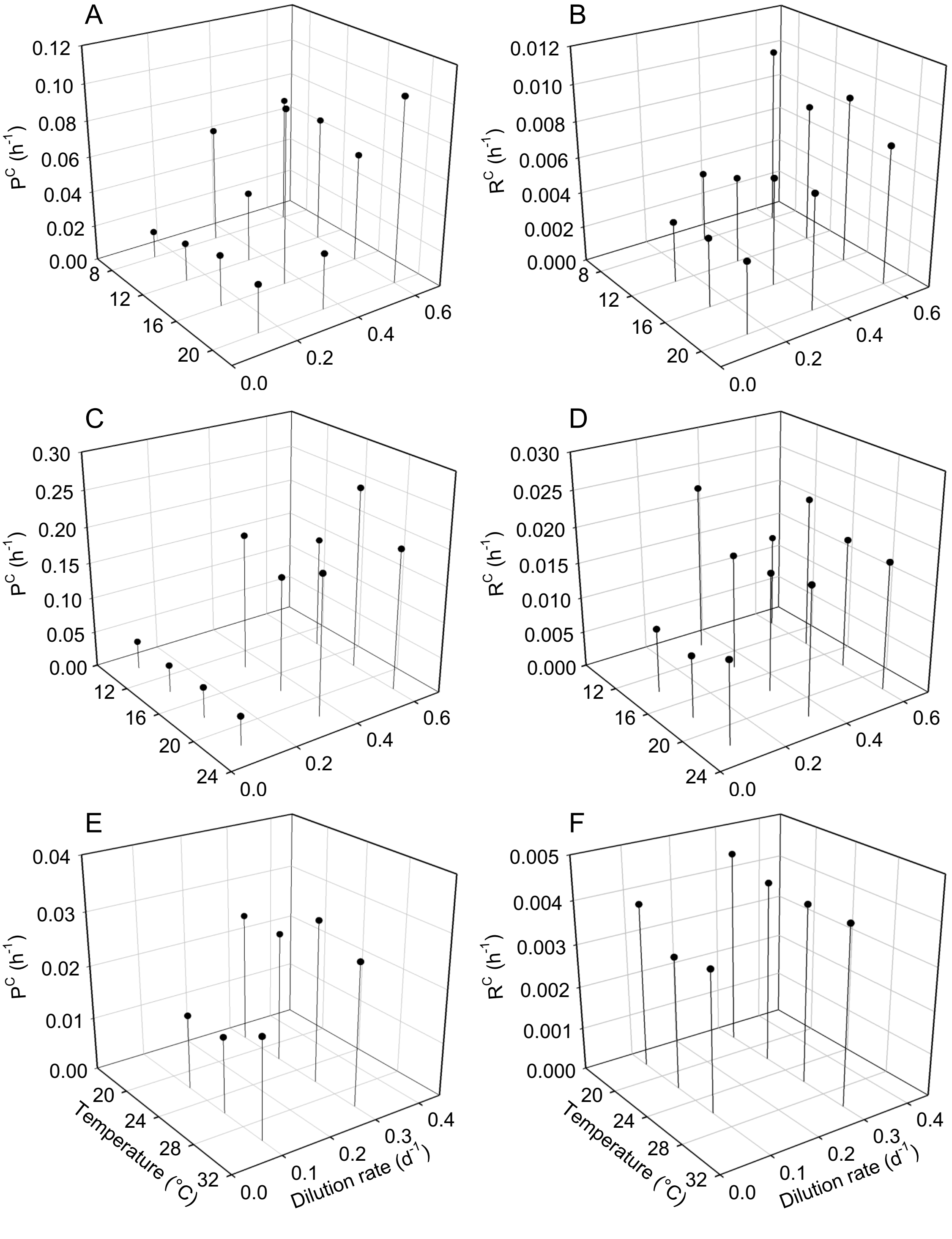

Fig. S7. Carbon-specific photosynthesis (P^C) and respiration (R^C) in *S. costatum* (top), *E. huxleyi* (middle) and *Synechococcus* (bottom) under different temperatures and dilution rates. Left: carbon-specific photosynthesis; right: carbon-specific respiration.

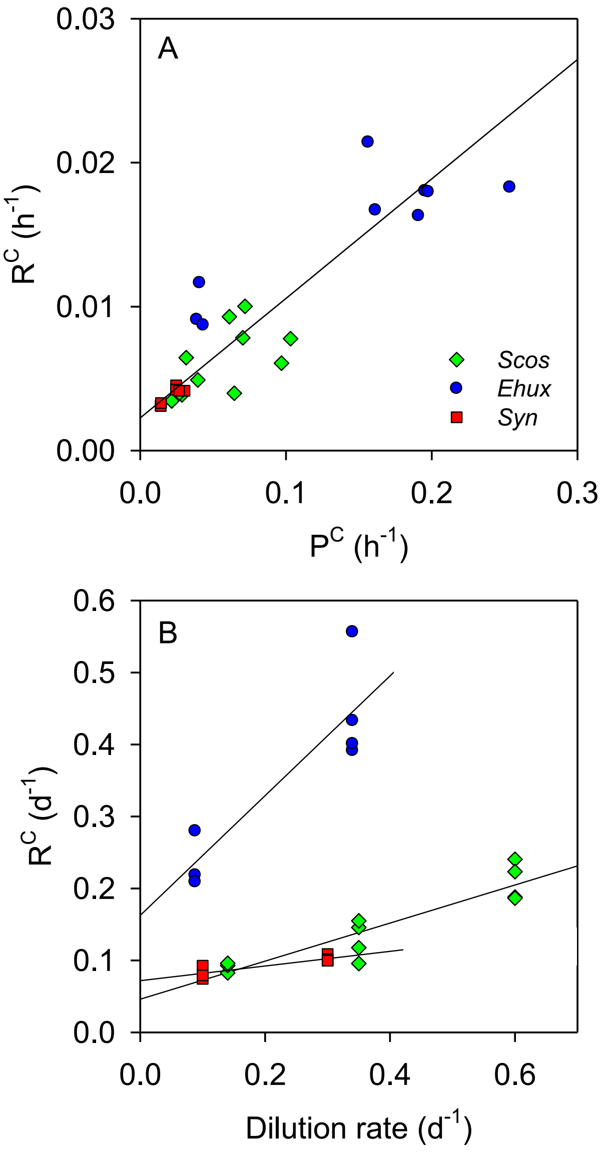

Fig. S8. A) Relationship between hourly rates of carbon-specific photosynthesis (P^C) and respiration (R^C) in *S. costatum*, *E. huxleyi* and *Synechococcus* growing at different temperatures and dilution rates. The linear fit (reduced major axis regression) is $y = 0.083\ x + 0.0023$ ($r^2 = 0.81$, $p < 0.0001$, $n = 26$). B) Relationship between dilution rate and daily R^C. Daily R^C was computed as hourly $R^C \times 24$. Lines are the OLS regression fits. The *y*-intercepts, which represent the basal metabolic rate, are 0.046 (SE = 0.017; 95% CI = 0.008, 0.084) for *S. costatum*, 0.163 (SE = 0.051; CI = 0.032, 0.293) for *E. huxleyi* and 0.072 (SE = 0.006; CI = 0.056, 0.088) for *Synechococcus*.

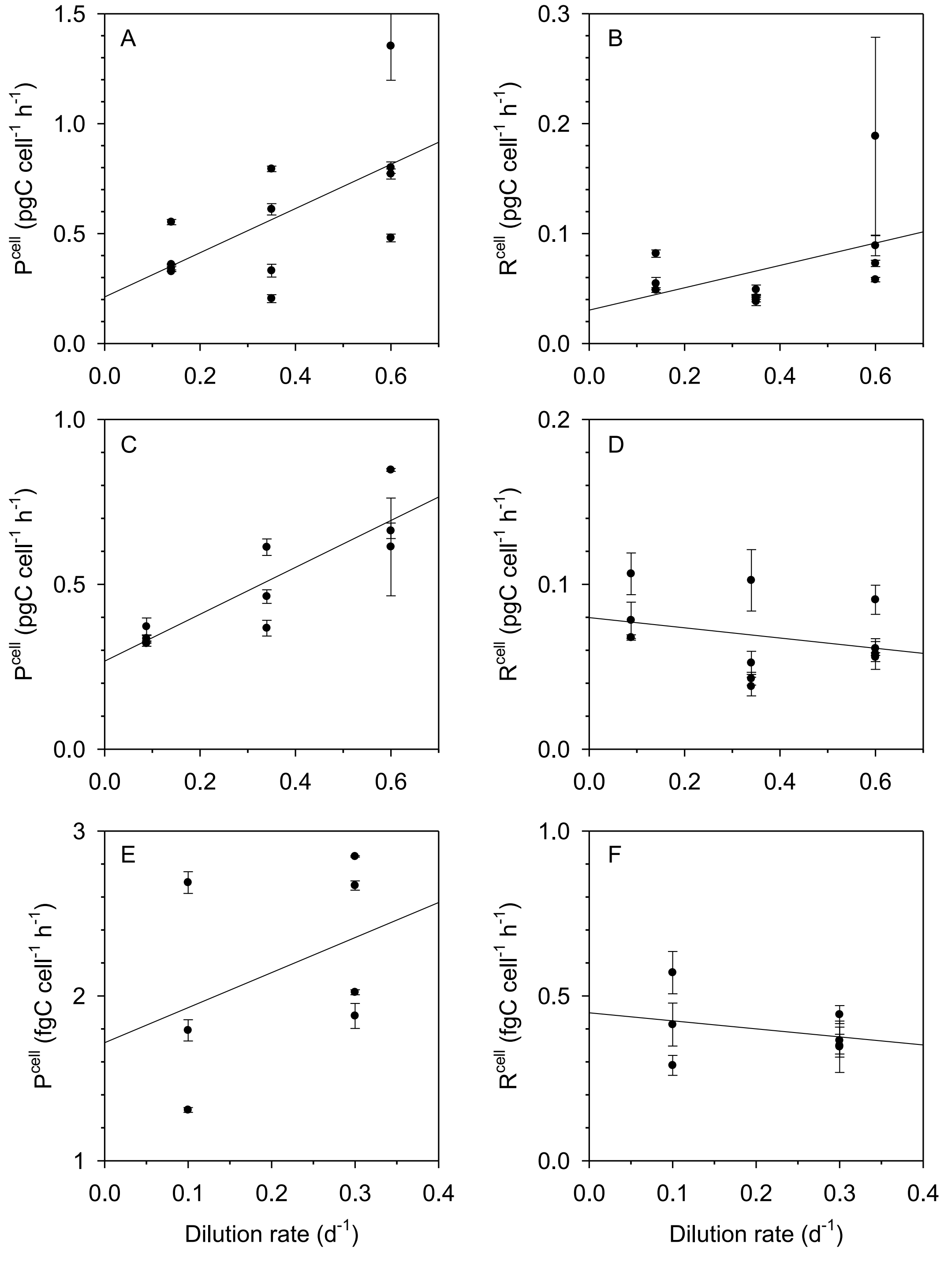

Fig. S9. Relationship between dilution rate and cell-specific rates of (left) photosynthesis and (right) respiration in A,B) *S. costatum*, C,D) *E. huxleyi* and E,F) *Synechococcus*. Bars indicate standard deviation and lines are the OLS regression fits.

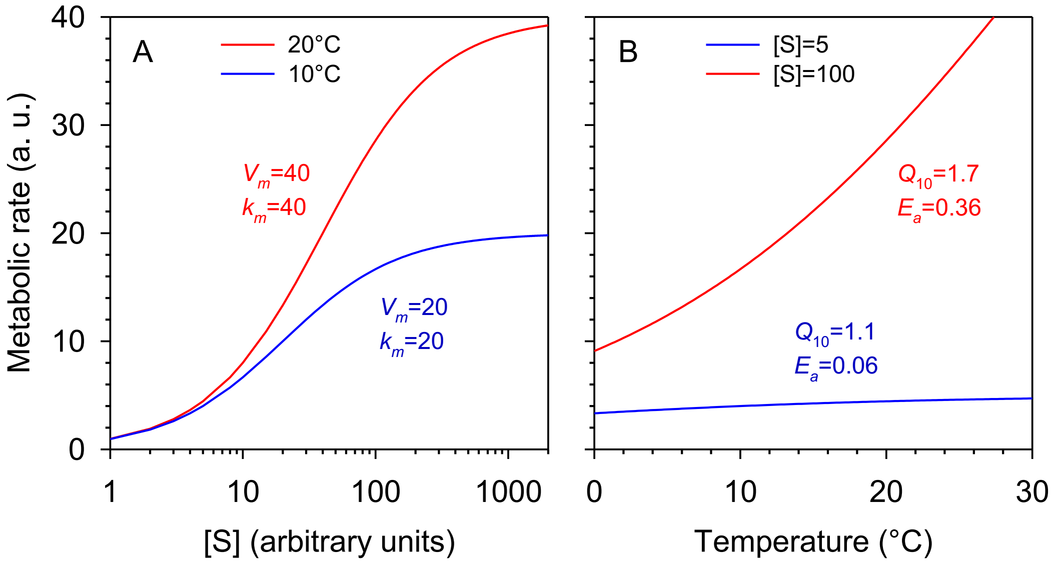

Fig. S10. Idealised example of the interaction between temperature and resource availability in the control of enzymatic kinetics and metabolic rates. Metabolic rate (V) is calculated according to Michaelis-Menten kinetics as $V=V_m[S]/([S]+k_m)$ where V_m is the maximum, nutrient-saturated rate, [S] is the substrate concentration, and k_m is the Michaelis-Menten or half-saturation constant. A) Relationship between [S] and V at 10°C and 20°C assuming that both V_m and k_m double with a 10°C increase in temperature (Q_{10} = 2). B) Relationship between temperature and V for a low ([S]=5) and a high ([S]=100) concentration of substrate, assuming that both V_m and k_m have a Q_{10} of 2 (equivalent to E_a=0.49 eV). The resulting temperature dependence of metabolic rate is much stronger under high (Q_{10}=1.7, E_a=0.36) than under low (Q_{10}=1.1, E_a=0.06) substrate concentration.
